# In hypertensive individuals, sleep time and sleep efficiency did not affect the number of angina episodes: a cross-sectional study

**DOI:** 10.1038/s41598-022-20255-y

**Published:** 2022-09-29

**Authors:** Ahmad H. Alghadir, Masood Khan, Mohammed Mansour Alshehri, Abdulfattah S. Alqahtani, Mishal Aldaihan

**Affiliations:** 1 Department of Rehabilitation Sciences, College of Applied Medical Sciences, King Saud University, Riyadh, Saudi Arabia; 2 Physical Therapy Department, Jazan University, Jazan, Saudi Arabia

**Keywords:** Cardiology, Diseases, Health care

## Abstract

Previous studies have reported adverse effects of short and long sleep duration on cardiovascular health. However, how sleep time and sleep efficiency affect angina have not been studied in hypertensive individuals. This study aimed to assess the relationship of sleep with angina. Using a cross-sectional design, data from 1563 hypertensive individuals were collected from the parent Sleep Heart Health Study (SHHS). Age, alcohol use, average diastolic blood pressure (ADBP), average systolic blood pressure (ASBP), cigarette use, sleep time, sleep efficiency, percent time in stage N3 of sleep, and body mass index (BMI) were used as covariates. Multiple linear regression, the Chi-Square test, and Pearson’s correlation coefficient were used for data analysis. Unadjusted sleep efficiency, sleep time, ADBP, and age were significant (p < 0.05) predictors of the number of angina episodes (Angina_n_). When the covariates were adjusted, only ADBP and ASBP were significant (p < 0.05) predictors of Angina_n_. Sleep efficiency, BMI, ADBP, sleep time, and age had a significant (p < 0.05) correlation with Angina_n_. In hypertensive individuals, sleep time and sleep efficiency did not affect Angina_n_ when adjusted for covariates. ADBP and ASBP were found to be significant predictors of Angina_n_ when the covariates were adjusted.

## Introduction

To maintain good emotional, mental, and physical health, individuals are required to have good sleep^1^. Several advantages of good sleep are reported. Normal sleep reduces the workload on the cardiovascular system, therefore, it may enhance cardiovascular longevity, and insufficient sleep may cause adverse consequences^2^.

Reduction in blood pressure (BP) is reported during normal sleep, where systemic BP decreases by an average of 5–10% in stages N1 and N2 of non-rapid eye movement (NREM) sleep and by 10–15% in stage N3^3,4^. The reduction in heart rate is also observed by 5–10% during NREM sleep. During rapid eye movement (REM) sleep, greater variability is observed in both BP and heart rate. Cardiac output also decreases during NREM sleep^4–6^.

Therefore, there are several benefits of nocturnal sleep on the cardiovascular system, especially through the reduction in systemic BP. Thus, if total sleep time is reduced due to any disorder then the cardiovascular system will be deprived of several benefits it accrues from good nocturnal sleep. The prevalence of sleep problems has been reported from 1.6 to 56% in several studies conducted in different countries throughout the world^7–9^. It’s reported that the ability to perform daily activities of living is directly affected in individuals having sleep disturbances^10^, which in turn induces a sedentary lifestyle^11^ that ultimately affects the health and quality of life of such individuals^12,13^. Sleep for less than 7 h duration is reported to be a significant risk factor for disorders of the nervous system, immune system, endocrine system, and cardiovascular systems, and may result in substance abuse, mood disorders, anxiety disorders, hypertension, cardiovascular diseases, impaired glucose tolerance, diabetes, and obesity in children and adults, thereby increasing the rate of mortality^14–19^. Asplund^20^ in a large epidemiological study of elderly people in Sweden reported that poor sleep was associated with cardiac dysrhythmias and angina pectoris.

Therefore, several studies have reported how sleep problems are related to cardiovascular system problems, however, to our knowledge, no previous study has been performed to test the association of the number of angina episodes (Angina_n_) with sleep in hypertensive individuals. The present study used data from the Sleep Heart Health Study (SHHS), which was initiated in 1994 as a multicenter prospective cohort study. The SHHS was a community-based longitudinal study that consisted of several ongoing cohort studies of the respiratory and cardiovascular systems^21^. Therefore, this study aimed to assess the association of Angina_n_ with sleep time and sleep efficiency in hypertensive individuals.

## Materials and methods

A cross-sectional design was used. The demographic characteristics (gender, age, body mass index (BMI), cigarette smoking, alcohol use), cardiovascular variables [average diastolic blood pressure (ADBP), average systolic blood pressure (ASBP), presence of angina, Angina_n_] and sleep variables (sleep time, percent time in stage N3 of sleep, percentage of time in bed that was spent sleeping) were obtained from the data collected by the parent study Sleep Heart Health Study (SHHS). Only participants of age 40 years or older at the time of the sleep study participated.

The specific aims, designs, and protocols of the SHHS have been previously described^21,22^. The SHHS Manual of Operations contains a comprehensive description of the methods used in study^23^. The primary aim of SHHS was to study the cardiovascular consequences of sleep-disordered breathing. The baseline data for SHHS were obtained from nine ongoing epidemiologic studies of cardiovascular and respiratory disease from December 1995 to January 1998 in diverse populations in the United States.

Only the data of hypertensive individuals were used from the SHHS. In SHHS, hypertension was defined as a resting BP of at least 140/90 mmHg or the use of antihypertensive medication^24^. The parent study (SHHS) included a baseline examination of 5804 participants, of which 3326 were not qualified as hypertensive and a total of 2478 participants qualified as hypertensive. However, out of 2478 participants, 915 participants’ data were missing for one or more variables. These participants were excluded from data analysis. Therefore, ultimately 1563 participants were used for data analysis (Tables 1 and 2). All methods were performed in accordance with the relevant guidelines and regulations.

**Table 1:** Descriptive statistics for participant’s characteristics and dependent and independent variables (total n = 1563, missing data n = 21).

	Normal (n = 1331) Mean ± SD	Angina present (n = 211) Mean ± SD	Total (1542) Mean ± SD/frequency (percentage)
Sleep efficiency (%)	49.2 ± 38.10	41.82 ± 38.84	48.19 ± 38.28
BMI (kg/m^2^)	25.57 ± 10.75	24.66 ± 10.71	25.44 ± 10.75
ADBP (mmHg)	63.41 ± 24.50	58.44 ± 24.19	62.73 ± 24.51
ASBP (mmHg)	119.04 ± 45.48	116 ± 47.05	118.63 ± 45.70
Sleep N3 (%)	9.79 ± 11.56	7.37 ± 11.05	9.46 ± 11.51
Alcohol use (n)	2.47 ± 6.23	2.7 ± 6.40	2.5 ± 6.25
Cigarette packs per year (n)	12.36 ± 19.45	20.49 ± 26.43	13.47 ± 20.73
Sleep time (minutes)	233.5 ± 184.39	197.09 ± 186.46	228.52 ± 185.04
Age (years)	71.71 ± 9.60	73.8 ± 9.22	71.99 ± 9.58
Male (n)			722 (46.2)
Female (n)			841 (53.8)

BMI: body mass index; SD: standard deviation; ADBP: average diastolic blood pressure; ASBP: average systolic blood pressure; Sleep N3: percent time in stage N3 of sleep.

**Table 2:** Median and percentile values of ADBP and ASBP.

		ADBP (mmHg)			ASBP (mmHg)		
		Normal (n = 1331)	Angina present (n = 211)	Total (1542)	Normal (n = 1331)	Angina present (n = 211)	Total (1542)
Median		70.00	65.00	69.00	129.00	127.00	129.00
Percentile	25	60.00	59.00	60.00	115.00	111.00	114.00
	50	70.00	65.00	69.00	129.00	127.00	129.00
	75	78.00	72.00	77.00	142.00	140.00	142.00

ADBP: average diastolic blood pressure; ASBP: average systolic blood pressure.

In SHHS, information related to covariates such as medical history and health-related characteristics was obtained using a standardized questionnaire through an interview by the study technician while visiting participants’ homes. The information regarding cardiovascular health was obtained by asking questions such as if the doctor ever told the participant that he had angina, etc. The responses of the participants were obtained in the form of ‘yes’, ‘no’, or ‘unsure’. Other related information like alcohol use or cigarette packs per year, was also obtained through interviews. Bodyweight, height, and BP were measured during the home visits of the participants using standard protocols. Bodyweight was measured by a portable scale. A manual of operation of SHHS contains detailed information regarding the protocols for these measurements^23^.

### BP measurement

The participants were asked to rest for 5 min, then in a sitting position, 3 BP readings were taken with a 5-min interval in between each reading, with the use of a mercury gauge sphygmomanometer. Systolic and diastolic BPs were measured to the nearest 2 mmHg^24^.

### Measurement of sleep variables

Sleep variables were measured using a single overnight EEG-based polysomnography at the participant’s home and in some cases where the home environment was not conducive to polysomnography, a non-home environment (e.g. a motel) was used^21^. The Compumedics P Series System (Abbotsford, Victoria, Australia) was used for polysomnography. During the evening home visit, this equipment was calibrated and sensors were placed on the participants^21^.

The institutional review board of each participating center approved the study. The signed informed consent was obtained from each participant. Data on smoking were also obtained through a questionnaire. Data regarding cardiovascular disease was also obtained through a questionnaire.

### Data analysis

SPSS statistical software version 26 (SPSS Inc., Chicago, IL, USA) was used for data analysis. A total of 1563 participants’ data was analyzed. The descriptive statistics of the outcome variable (Angina_n_) and predictors (age, alcohol use, ADBP, ASBP, cigarette smoking, sleep time, sleep N3, BMI, sleep efficiency) are shown in Table 1. Multiple linear regression analysis was performed to predict the relationship of the dependent variable, i.e. Angina_n_, with predictors. Pearson’s correlation coefficient was used for correlational analysis between Angina_n_ and other covariates. A Chi-square test was performed to test the relationship between categorical variables (presence of angina and gender). The confidence interval was established at 95%, p < 0.05 considered significant.

Variables:

- Alcohol use: number of alcoholic drinks per day.
- Cigarette smoking: Cigarette packs smoked per year.
- Sleep time: sleep time when the entire sleep was captured.
- Sleep N3: percent time in stage N3 (stages 3 and 4 according to traditional terminology^25^) of sleep.
- Sleep efficiency: percentage of time in bed that was spent sleeping, or the ratio of total sleep time to total time in bed, expressed as a percentage.

## Results

Multiple linear regression revealed that unadjusted sleep efficiency, sleep time, ADBP, and age were significant (p < 0.05) predictors of Angina_n_ and were responsible for 0.3%, 0.8%, 0.2%, and 0.9% variance in Angina_n_, respectively, in the hypertensive population (Table 3). When all the covariates (age, alcohol, ADBP, ASBP, cigarette smoking, sleep efficiency, sleep time, sleep N3, and BMI) were adjusted then only ADBP and ASBP were found to be significant predictors of Angina_n_ (p < 0.05). The adjusted covariates had a significant impact (p < 0.01) on Angina_n_ and were responsible for 2.3% of the variance (Table 4). Pearson’s correlation coefficients between Angina_n_ and other predictors are presented in Table 5. Sleep efficiency, BMI, ADBP, sleep time, and age were found to have a significant (p < 0.05) correlation with Angina_n_. Among these significant correlations, only age had a positive correlation, otherwise, the rest of the covariates had a negative correlation. Pearson correlation coefficient values are presented in Table 5. The Chi-square test revealed a significant difference (p = 0.01, Cramer’s V = 0.066) in the presence of angina with gender differences (male and female) (Table 6). The presence of angina was found to be significantly (p < 0.05) greater in the male hypertensive population than in the female hypertensive population.

**Table 3:** Multiple linear regression analysis.

Unadjusted for predictors							Adjusted for predictors		
Covariate	R-value	Adjusted R-square value	Un-standardized beta coefficient	F	t-value	*p*-value	Standardized coefficients beta	t-value	*p*-value
Sleep efficiency	0.061	0.003	− 0.001	5.895	− 2.428	0.015*	− 0.123	− 1.125	0.261
BMI	0.043	0.001	− 0.003	2.857	− 1.690	0.091	0.041	1.044	0.297
ADBP	0.095	0.008	− 0.003	14.230	− 3.772	0.000*	− 0.307	− 4.916	0.000*
ASBP	0.029	0.000	0.000	1.323	− 1.150	0.250	0.224	3.543	0.000*
Sleep N3	0.039	0.001	− 0.002	2.364	− 1.537	0.124	0.007	0.209	0.834
Alcohol use	0.004	− 0.001	− 0.001	.028	− 0.169	0.866	− 0.005	− 0.194	0.846
Cigarette packs per year	0.031	0.000	0.001	1.500	1.225	0.221	0.025	0.965	0.335
Sleep time	0.055	0.002	0.000	4.822	− 2.196	0.028*	0.091	0.870	0.384
Age	0.097	0.009	0.008	14.915	3.862	0.000*	0.043	1.527	0.127
Gender	0.016	0.000	− 0.023	0.380	− 0.616	0.538			

Association of the number of angina episodes with sleep efficiency, BMI, average diastolic blood pressure, average systolic blood pressure, percent time in stage N3 of sleep, alcohol use, cigarette use, sleep time, age, and gender.

BMI: body mass index; ADBP: average diastolic blood pressure; ASBP: average systolic blood pressure; Sleep N3: percent time in stage N3 of sleep.

*Significant.

**Table 4:** Model summary for adjusted predictors (dependent variable: number of angina episodes; predictors (constant): age, alcohol use, average diastolic blood pressure, average systolic blood pressure, cigarette use, percent time in stage N3 of sleep, sleep time, BMI and sleep efficiency).

R	Adjusted R square	F change	df1	df2	*p*-value F change	*p*-value ANOVA
0.168	0.023	4.996	9	1553	0.000*	0.000*

**Table 5:** Pearson’s coefficient of correlations (R) of different covariates with the number of angina episodes.

	Pearson’s correlation coefficient (R)	*p*-value
Sleep efficiency	− 0.061	0.008*
BMI	− 0.043	0.046*
ADBP	− 0.095	0.000*
ASBP	− 0.029	0.125
Sleep N3	− 0.039	0.062
Gender	− 0.016	0.269
Alcohol use	− 0.004	0.433
Cigarette packs per year	0.031	0.110
Sleep time	− 0.055	0.014*
Age	0.097	0.000*

BMI: body mass index; ADBP: average diastolic blood pressure; ASBP: average systolic blood pressure; Sleep N3: percent time in stage N3 of sleep.

*Significant.

**Table 6:** Cross-tabulation for the presence of angina vs gender and chi-square test result (n = 1542).

Presence of Angina	Gender		Total	Pearson Chi-Square *p*-value	The effect size for Chi-Square (Cramer’s V)
	Male	Female			
No	598	733	1331	0.010*	0.010
Yes	115	96	211		
Total	713	829	1542		

*Significant.

## Discussion

The results of the present study revealed that unadjusted sleep efficiency, sleep time, ADBP, and age were significant predictors of Angina_n_ in hypertensive individuals. When all covariates (age, BMI, alcohol use, ADBP, ASBP, cigarette smoking, sleep time, sleep N3, and sleep efficiency) were adjusted, then only ADBP and ASBP were found to be significant predictors of Angina_n_. Adjusted covariates were found to be responsible for only 2.3% of the variance in Angina_n_. This indicates that there will be several factors other than the above-mentioned covariates that can predict more variation in the Angina_n_. The present study aimed to know the association of sleep time and sleep efficiency with Angina_n_, which has not been found when sleep time and efficiency were adjusted to other covariates. Therefore, in the present study, hypertension played a major role in predicting the Angina_n_ rather than sleep time and efficiency.

Previous epidemiological studies have shown that impaired sleep patterns like long and short sleep duration are related to several cardiometabolic impairments such as hypertension, diabetes, obesity, hypercholesterolemia, stroke, and myocardial infarction^26,27^.

In the present study, it should be noted that the mean sleep efficiency was 48.33%, which is quite low. Several factors could be responsible for low sleep efficiencies, such as participants belonging to the older age group (mean age 72.03 years), the presence of hypertension, and other cardiovascular diseases in the study cohort. A study performed by Didikoglu et al. showed a relationship between old age and reduced sleep efficiency. They reported an 18.6% decrease in sleep efficiency in older adults between the age of 40 and 100 years^28^.

Sleep duration is associated with cardiovascular risks^29^, and in particular, with an increased risk of myocardial infarction^30^ and it is the combination of poor sleep quality and short sleep duration that causes maximum risks^31^. A study by Buxton and Marcelli^32^ reported that both short and long durations of sleep were predictors of cardiovascular disease.

Reduced sleep duration has been reported to result in a general increase in inflammatory markers, reduced variability in heart rate, increased BP, disrupted hypothalamic axis, glucose intolerance, hyper-activation of the sympathetic nervous system, and increased cortisol levels^33–37^. Other studies found that the risk of obesity and weight gain is more in individuals with poor sleep quality or short sleep duration^38,39^. However, no study in our knowledge reported the effects of sleep duration and efficiency on angina in hypertensive individuals. Therefore, the comparison of the findings of the present study with other studies is difficult.

However, a systemic review and meta-analysis performed by Cappuccio et al. found that sleep of short duration was not associated with an increased risk of developing cardiovascular diseases or dying from it however they reported that sleep of long duration was associated with an increased risk of developing cardiovascular disease or dying from it^40^. The reason for the association of long duration of sleep with CVD could be because other factors such as subclinical diseases, physical inactivity, low socioeconomic status, and depression are associated with long duration of sleep and thus may confound the associations of long duration of sleep with CVD^41,42^. Thus long sleep durations may even be the consequence of comorbidities^40,41^.

The findings of the present study are also supported by the study of Qureshi et al.^43^ which reported that sleep durations of fewer than 6 h and more than 8 h were not associated with an increased risk of coronary artery disease.

The results of the present study indicated that the presence of angina is greater in men compared to women. According to the study by Maas et al.^44^, women develop cardiovascular diseases 7–10 years later than men. Before menopause, women have a low coronary heart disease event rate^45^. The reason for this may be that women have premenopausal protection against ischemic heart diseases^46^. Endogenous estrogen hormone released before menopause is assumed to delay the manifestation of atherosclerotic diseases in women^44^. Similar findings were reported by Hemingway et al. who reported that the age-standardized annual incidence per 100 population of all cases of angina was higher in men than in women^47^. Additionally, a study by Murphy et al. reported that angina is more common in men than in women^48^.

The present study revealed that, when unadjusted, age is a significant predictor of Angina_n_ and was responsible for 0.9% variance. Age was also found to have a significant positive correlation, although small (r = 0.097), with Angina_n_. But when adjusted for other covariates, no significant impact of age was found on Angina_n_. However contrary findings were reported by the study of Fisher et al., which reported that older people were more likely to have angina and more severe symptoms when compared with people with a similar extent of disease^49^. They reported that age is an independent and significant predictor of the presence and severity of angina, even after adjusting for covariates. Several factors may explain the association of aging with the presence of angina, such as deconditioning due to a sedentary lifestyle, physiological factors related to aging, and the disease process^49^.

An interesting finding from the present study is that no association was found between the number of cigarette packs used in a year and Angina_n_. Similar findings were reported by Pujades-Rodriguez^50^ who reported no differences in lifetime risks for stable angina according to the smoking status. However, a study by Merry et al.^51^ reported that smoking increased the risk of unstable angina. Also, the study by Wilmink et al.^52^ found a relative risk of 1.3 for angina for current smokers compared to non-smokers.

This study used gold standard measurements for sleep parameters and cardiovascular health. However, some limitations are needed to be mentioned for future studies. Cardiovascular medication adherence was not controlled, which has an impact on BP and cardiovascular health. Depression and anxiety symptoms increase during aging which has an impact on overall sleep quality. Future studies are needed to assess the impact of sleep on cardiovascular health with controlled psychological symptoms. Future studies should control extraneous variables like physical activity and sedentary lifestyle because they affect cardiovascular health.

## Conclusion

In hypertensive individuals, sleep time and sleep efficiency did not affect Angina_n_ when adjusted for covariates. Average diastolic and systolic BP were found to be significant predictors of Angina_n_ when covariates were adjusted.

## Data Availability

The datasets generated and/or analyzed during the current study are not publicly available due but are available from the corresponding author on reasonable request.

## Abbreviations

Angina_n_: Number of angina episodes
BMI: Body mass index
REM: Rapid eye movement
NREM: Non-rapid eye movement
BP: Blood pressure
ADBP: Average diastolic blood pressure
ASBP: Average systolic blood pressure
SHHS: Sleep heart health study
